# Prevalence and characteristics of treatments for sleep disordered breathing in people receiving dialysis: a scoping review

**DOI:** 10.1007/s40620-025-02370-x

**Published:** 2025-09-09

**Authors:** Daniel S. March, Lizelle Bernhardt, Kelly Barber, Ffion Curtis, Patrick J. Mowles, Nina Morris, Ellesha A. Smith, Sonny Vargeson, James O. Burton

**Affiliations:** 1 Department of Cardiovascular Sciences, University of Leicester, John Walls’ Renal Unit, University Hospitals of Leicester NHS Trust, Leicester, UK; 2 NIHR Leicester Biomedical Research Centre, University of Leicester and University Hospitals of Leicester NHS Trust, Leicester, UK; 3 Department of Cardiovascular Sciences, University of Leicester, Leicester, UK; 4 Leicestershire Partnership NHS Trust, Leicester, UK; 5 Faculty of Health, Liverpool John Moores University, Liverpool, UK; 6 Liverpool Reviews and Implementation Group, Institute of Population Health, University of Liverpool, Liverpool, UK; 7 Leicester Medical School, University of Leicester, Leicester, UK; 8 Department of Population Health Sciences, University of Leicester, Leicester, UK

**Keywords:** Dialysis, Scoping review, Meta-analysis, Sleep apnoea, Continuous positive airway pressure

## Abstract

**Background:**

Individuals with kidney failure experience elevated cardiovascular risk, potentially worsened by the presence of sleep disordered breathing. Despite this association, prevalence of sleep apnoea, and evidence for effective treatments are poorly understood in people with kidney failure. This review examines sleep apnoea prevalence, types of sleep apnoea, and treatment interventions in people with kidney failure receiving dialysis.

**Methods:**

Guidelines for scoping reviews were followed and the following databases were searched for both peer reviewed and grey literature: MEDLINE, EMBASE, CINAHL, Cochrane Central Register of Controlled Trials (CENTRAL), ClinicalTrials.gov, the Web of Sciences Core Collection, OpenGrey, ETHos and ProQuest. All databases were searched from inception to 18th October, 2024. Random-effects proportional meta-analysis was used to estimate prevalence. A narrative synthesis of the interventions from the included studies for sleep apnoea was reported.

**Results:**

There were 36 included studies. Pooled data from 19 studies indicated that sleep apnoea prevalence was 59% (95% CI 47%, 70%). Pooled data estimated mild apnoea prevalence at 21% (95% CI 16%, 26%) (11 included studies), with moderate and severe prevalence being 44% (95% CI 30%, 60%) (14 included studies). The majority of sleep apnoea was obstructive (75% (95% CI 53%, 89%)) with the remaining being central (15% (95% CI 8%, 28%)) and mixed (15% (95% CI 3%, 49%)) in nature.

**Conclusion:**

The prevalence of sleep apnoea is high in people receiving dialysis. Currently there is insufficient evidence for the effective treatment of sleep apnoea in this population.

**Graphical Abstract:**

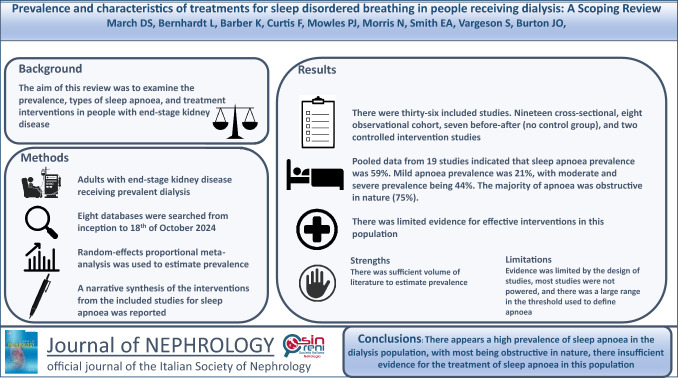

**Supplementary Information:**

The online version contains supplementary material available at 10.1007/s40620-025-02370-x.

## Introduction

Individuals with kidney failure have a significantly increased risk of cardiovascular disease, which is the leading cause of death in this population. Despite improvements in the care and management of these individuals, strategies to mitigate this enhanced cardiovascular risk have had little impact due to the preponderance of non-traditional risk factors (such as chronic inflammation, uraemia, anaemia, chronic kidney disease-mineral and bone disorder, and haemodialysis-induced myocardial stunning) in this population [1–3]. One unrecognised factor (possibly due to different clinical presentation [4]) that may contribute to this enhanced cardiovascular risk is the presence of sleep disordered breathing [5]. Sleep disordered breathing is characterised by periodic interruptions in breathing during sleep with consequent adverse effects on the cardiovascular system mediated through chemical, autonomic, mechanical and inflammatory pathways. The main subgroups of sleep disordered breathing include obstructive sleep apnoea, central sleep apnoea, and mixed sleep apnoea [6]

Prevalence of sleep apnoea is believed to increase as chronic kidney disease (CKD) progresses [7], and has been reported to be highest in those with kidney failure [7], with the majority believed to be obstructive in nature [7–9]. Sleep apnoea has been associated with cardiovascular, and all-cause mortality in CKD [8–11]. A prospective cohort study of 558 incident dialysis patients reported the risk of sudden cardiac death was the highest in those individuals with obstructive sleep apnoea, after controlling for known cardiovascular risk factors [12].

A recent systematic review [13] reported that prevalence of sleep apnoea was 56% (95% confidence interval (CI) 0.42%, 0.69%) in people with kidney failure. However, this review was limited by the fact that the pooled analysis included data from studies that had used different thresholds to define sleep apnoea, and included both incident and prevalent dialysis patients, as well as kidney transplant recipients [13]. In both the general and kidney failure population, continuous positive airway pressure (CPAP) is the treatment of choice for clinically significant moderate to severe obstructive sleep apnoea. However, it is unclear whether there is any evidence to support this treatment within this population. Therefore, the aims of this scoping review were to; (1) provide a summary of the prevalence of sleep apnoea in the dialysis population; (2) provide a summary of the prevalence of obstructive versus central sleep apnoea in the dialysis population; (3) provide a summary of current treatment interventions for sleep apnoea in the dialysis population.

## Materials and methods

A scoping review was selected to examine the prevalence and treatment interventions for sleep apnoea in the context of the dialysis population, within all available literature. We followed the preferred reporting items for systematic reviews and meta-analyses extension for scoping reviews (PRISMA-ScR) checklist [14] (Table S1), and the Arksey & O’Malley [15] framework for conducting this review. This review was prospectively registered on Figshare: https://figshare.com/articles/online_resource/ESKD_and_SDB_Scoping_Review_Protocol_updated_v1_2_18_05_2024/25853539. The last protocol update was on 18th May, 2024.

### Inclusion criteria

The inclusion criteria were:

- Participants: Individuals (over the age of 18 years) with kidney failure receiving prevalent dialysis as kidney replacement therapy, and without a previous diagnosis of co-existing sleep disordered breathing (central or obstructive sleep apnoea). Individuals had to be unselected (e.g. with no history of sleep disordered breathing or disturbances and not previously pre-screened with sleep questionnaires or oximetry testing).
- Concept: This review considered studies that included objective assessment of sleep disordered breathing (in-lab or home polysomnography and home sleep apnoea testing such as polygraphy) and any treatment interventions (positive airway pressure, mandibular advancement devices, positional modifiers, surgery, or lifestyle advice) for sleep disordered breathing (central, obstructive and mixed sleep apnoea). Due to the poor detection of sleep disordered breathing in this population using screening methods alone, studies that had solely used such questionnaires (e.g. STOP-Bang questionnaire or Berlin questionnaire) or oximetry testing, were excluded. Studies that had used codes (such as ICD-10) to define sleep apnoea were also excluded. Studies had to report on the prevalence of sleep apnoea, or include an intervention for apnoea.
- Setting: Individuals living with kidney failure and co-morbid sleep apnoea may present in several clinical settings including outpatient clinics or haemodialysis units.
- Types of study: This scoping review considered the following study designs (but not limited to): interventional studies (randomised and non-randomised controlled trials, before-after (pre-post) studies), observational studies (e.g. cohort, cross-sectional, case control, and case series), and qualitative studies relating to sleep disordered breathing. Published protocols of studies and conference abstracts were included.

### Search strategy

The following bibliographical databases and trial registers were searched for completed and ongoing studies: MEDLINE, EMBASE, CINAHL Plus, Cochrane Central Register of Controlled Trials (CENTRAL), ClinicalTrials.gov, and the Web of Sciences Core Collection. Detailed individual search strategies, with appropriate truncation and word combinations, were developed for each database. OpenGrey, ETHos and ProQuest were searched for unpublished data. All databases were searched from inception to 18th October, 2024, with no limits on language set. Database searches were supplemented with a Google Scholar search with the first 200 titles screened for inclusion [16]. An example of a full search strategy for MEDLINE is presented in Table S2.

### Study selection and data charting

Search results were compiled using the web-based screening and data extraction tool Rayyan (www.rayyan.ai). Duplicate citations were removed, and a two-part study selection process was used: (1) title and abstracts were screened independently by two reviewers against the inclusion criteria, and (2) full-text articles (not excluded based on title or abstracts) were retrieved and assessed by two reviewers. If there was a disagreement, then this was resolved through the inclusion of a third reviewer. Non-English reports deemed relevant at the title and abstract screening stage were included, decisions about including these reports were made on a case-by-case basis at the full-text screening stage [17]. Relevant conference abstracts were included at the title and abstract screening stage and those that provide data for pooling were included; if data were not provided then authors were contacted. If data could not be obtained then the abstract details were provided in the supplementary materials. When multiple publications were identified, we included the study with the information (pertaining to prevalence and/or interventions) required to answer the aims of the review.

### Data extraction and synthesis

We developed, tested, and refined a structured data collection form based on the Cochrane Data Extraction Template for interventions but with modifications for non-interventional studies. One reviewer undertook data extraction for each study, with a second reviewer (DSM or LB) cross-checking all extracted data. For each included study, characteristics including study details and design, kidney failure population (including demographic factors), sample size, sleep disordered breathing definitions, sleep recording parameters, intervention characteristics, prevalence data, and study main results were extracted and presented as text, tables and figures. Proportional meta-analysis was used to pool the proportions of participants with sleep apnoea out of the total number of participants in studies that classified sleep apnoea using the criteria apnoea-hypopnea index > 5 events per hour, or apnoea-hypopnea index ≥ 5 events per hour, respiratory disturbance index > 5, or disordered breathing events ≥ 5. The ‘metaprop’ command in the ‘meta’ package in R, through RStudio [18], was used to run random effects proportional meta-analysis models, using the inverse variance method and a logit transformation. Subgroup analyses for the severity of sleep apnoea (apnoea-hypopnea index > 5 or apnoea-hypopnea index ≥ 5 to < apnoea-hypopnea index 15 for ‘mild’; apnoea-hypopnea index ≥ 15 or respiratory disturbance index ≥ 15 for ‘moderate and severe’) and the types of sleep apnoea (central, obstructive or mixed) were performed. The chi-squared test was used to test whether the subgroup proportions were different. The between-study heterogeneities, ${\tau }^{2}$, and the proportions of variation due to heterogeneity of the total variation, ${I}^{2}$, are reported. For the meta-analyses reporting prevalence and type of sleep apnoea, only baseline data were taken (i.e. before any intervention or treatment) from any included before-after (pre-post) or controlled intervention study. We created a narrative synthesis of the findings from included studies structured around the interventions reported for the treatment of sleep apnoea. No data from the interventional studies were statistically synthesised.

### Quality assessment

Whilst quality assessment of the included studies is not an essential component of a scoping review, we chose to include it because we felt that it would provide value. Our rationale was to identify key considerations for the design of a future randomised controlled trial in this area. We assessed the quality of the full-texts of included studies (*n* = 33). The National Heart, Lung, and Blood Institute (National Institutes of Health) Study Quality Assessment Tools (available from: https://www.nhlbi.nih.gov/health-topics/study-quality-assessment-tools) was used. Table S3 reports the criteria used to assess the quality of the included studies. Two reviewers evaluated the quality of the included studies independently. A narrative synthesis of the quality of studies is provided against criteria outlined in the Study Quality Assessment Tool. We were unable to assess the quality of the three included conference abstracts due to insufficient information.

## Results

**Fig. 1:**
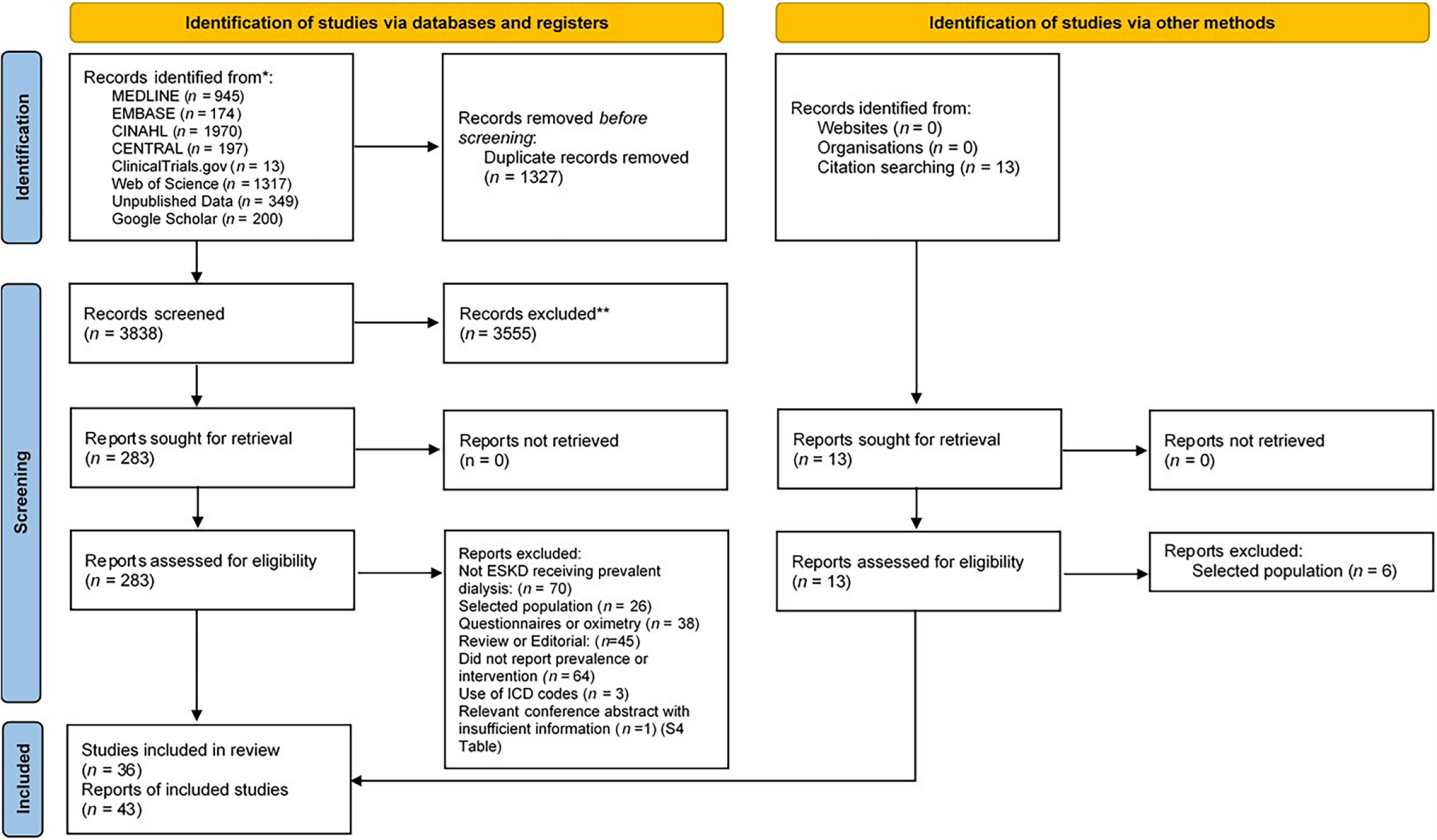
PRISMA 2020 flow diagram of study selection including searches of databases, registers and other sources. Three studies had two reports each which included the same participants with different outcomes [29, 45, 48, 51, 69, 70], of which we only included three earlier reports of the studies [29, 45, 48] to extract data on prevalence (see Table 1). Two further conference abstracts [71, 72] were later published as full text studies [41, 44] so we included the full texts reports of the studies and counted the abstracts as companion reports. Lastly a study report [26] included (and added to) data from participants that had been previously reported [73], therefore we included the study with the larger sample size [26]. Although not specifically stated, it appears that two reports contain the same participants [32, 74], therefore we have only reported data from the most recent [32]

Figure 1 provides a flow diagram of report identification and inclusion. We identified one conference abstract which appeared relevant, but limited data were suitable for synthesis so we excluded it (see Table S4). This left 43 individual reports of 36 separate studies reporting data on prevalence and treatments for sleep apnoea (Table 1). Within this group, we included two study reports [19, 20] which included individuals with either a history of sleep disorders [19], or who had been pre-screened with oximetry [20], (and therefore were not unselected) as we wanted to include a wide breadth of evidence for interventions to treat sleep apnoea in the kidney failure population. We did not utilise data from these studies [19, 20] to estimate prevalence of sleep apnoea.

**Table 1:** Characteristics of included studies

Study	Country	End stage kidney disease population	Sample size	Age; sex	Study design	Assessment device; sleep disordered breathing definition	Apopnea definition	Hypopnea definition	Scoring criteria	Primary aim of study
Beecroft et al., 2006 [63]	Canada	Haemodialysis and Peritoneal dialysis	*n* = 58	Apnoeic group: 50.7±11.3 years (*n* = 38); Non-apnoeic group: 43.2± 11.7; Apnoeic group: *n* = 28/38 (74%); Non-apnoeic group: *n* = 11/20 (55%)	Cross-sectional	Polysomnography; AHI ≥10	Absence of airflow >10 s	Reduction >10 s in respiratory effort to a value between 10 and 50 percent of the baseline level during sleep, with or without an associated decrease in oxygen saturation.	Rechtschaffen & Kales, 1968	Investigate association between sleep apnoea and chemo-responsiveness
Beecroft et al., 2008 [45]	Canada	Haemodialysis	*n* = 24	range = 32-68 y; *n* = 15 (63%)	Observational Cohort	Polysomnography; AHI ≥15	Absence of airflow >10 s	Reduction in respiratory effort for 10 s or more associated with an arousal and/or reduction in oxygen saturation >3%	Rechtschaffen & Kales, 1968	Investigate the association between sleep apnoea and both pharyngeal area and chemosensitivity
Chafekar et al., 2020 (Conference Abstract) [21]	India	Haemodialysis	*n* = 34	49.5±13.58 years; male *n* = 22 (64.7%)	Cross-sectional	Polysomnography; AHI ≥5	Not reported	Not reported	Not reported	Investigate the prevalence of sleep apnoea
Chu et al., 2020 [20]	Australia	Haemodialysis	*n* = 15	70±15; Male *n* = 12 (80%)	Controlled Intervention	Polysomnography; AHI ≥15	Not reported	Not reported	AASM 2007	Investigate the effect of hameodialfiltration versus haemodialysis on sleep apnoea.
Daabis & El-Gohary, 2012 [24]	Eqypt	Haemodialysis and Kidney Transplant Recipients	*n* = 55;* n* = 15 (haemodialysis)	34±5.66; *n* = 11 (73%)	Cross-sectional	Polysomnography; AHI ≥5	Not reported	Not reported	AASM 2007	Investigate the prevalence of sleep disordered breathing in haemodialysis versus kidney transplant recipients.
de Oliveira Rodrigues et al., 2005 [36]	Brazil	Haemodialysis and Peritoneal Dialysis	*n* = 45	37.1±10.9 years; *n* = 28/45 (62%)	Cross-sectional	Polysomnography AHI ≥5	Not reported.	Not reported.	Rechtschaffen & Kales, 1968	Investigate whether sleep apnoea is associated with clinical factors.
Hallett et al., 1995 [47]	Australia	Haemodialysis and Peritoneal Dialysis	*n* = 15	48±11; *n* = 9 (60%)	Cross-sectional	Polysomnography; RDI (threshold not defined)	Absence of airflow >10 s, or Absence of airflow >10 s with drop in SaO_2_ of more than 4% below baseline.	Reduced airflow or thoracoabdominal movement by more than 50% of baseline measurement for 10 s with undefined desaturation, or less than 10 s with a fall in SaO_2_ greater than 4%.	Group consensus	Investigate the prevalence of sleep disordered breathing.
Hanly & Pierratos, 2001 [29]	USA	Haemodialysis	*n* = 14	45±9; *n* = 10 (71%)	Before after (no control group)	Polysomnography; AHI >15	Absence of airflow >10 s	Reduction >10 s in the respiratory effort to a value between 10 and 50 percent of baseline level during sleep, with or without an associated decreasenin oxygen saturation.	Rechtschaffen & Kales, 1968	Investigate the effect of nocturnal haemodialysis on sleep apnoea.
Harmon et al., 2018 [30]	Brazil	Haemodialysis	*n* = 55	50±9; *n* = 27 (49%)	Observational cohort	Polysomnography; AHI ≥5	Absence of airflow >10 s	A reduction in airflow of ≤30% or thoracoabdominal movement for a at least 10 s with and oxygen desaturation ≥4%.	AASM 2007	Investigate variables associated with obstructive sleep apnoea.
Jean et al., 1995 [31]	France	Haemodialysis	*n* = 10	35-71 years; *n* = 8/10 (80%)	Controlled intervention	Polysomnography; 5 DBE/h	Absence of airflow >10 s	Decrease in airflow to less than one third of control for 10 seconds	Not reported	Investigate sleep disordered breathing following acetate or bicarbonate haemodialysis treatment
Jung et al., 2010 [32]	South Korea	Haemodialysis	*n* = 30	55.8±10.6; *n* = 21/30 (70%)	Observational cohort	Polysomnography; AHI >5	Absence of airflow >10 s	Partial reduction in respiratory airflow to less than 50% of the baseline level for more than 10 s with or without an associated fall in SaO_2_	Rechtschaffen & Kales, 1968	Investigate the associations between sleep disorders and mortality.
Jurado-Gamez et al., 2007 [75]	Spain	Haemodialysis	*n* = 32	54±16; *n* = 24 (75%)	Cross-sectional	Polysomnography; AHI >10	Absence of airflow >10 s	Defined as a drop in airflow of 50% from baseline lasting at least 10 s, accompanied by a dip in oxygen saturation ≥ 3%.	Rechtschaffen & Kales, 1968	Investigate sleep apnoea in people receiving haemodialysis
Jurado-Gámez et al., 2008 [49]	Spain	Haemodialysis	*n = *9	42±16 years; 7/9 (78%)	Before after (no control group)	Polysomnography; AHI ≥10	Absence of airflow >10 s	Defined as a drop in airflow of 50% from baseline lasting at least 10 s, accompanied by a dip in oxygen saturation ≥ 3%,	Rechtschaffen & Kales, 1968	Investigate the effect of kidney transplantation on sleep apnoea
Kang et al., 2022 [46]	South Korea	Peritoneal Dialysis	*n* = 103	56±11 years; *n* = 69 (67%)	Observational Cohort	Polysomnography; AHI >15	Absence of airflow >10 s	Hypopnea was defined as more than 10 s of abnormal respiratory event accompanied by more than 30% reduction in airflow from baseline and more than 4% decrease in oxyhemoglobin.	AASM 2007	Investigate the associations between sleep apnoea and volume status, and the effect on mortality
Koehnlein et al., 2009 [25]	Germany	Haemodialysis & Normal Kidney Function Controls	*n* = 30;* n* = 15 (haemodialysis)	62.9±13.8 years; *n* = 11/15 (73%)	Cross-sectional	Polysomnography; AHI ≥10	Absence of airflow >10 s despite continuing thoracic and abdominal movements.	Hypopnea was defined by a reduction of the oronasal airflow of more than 50%	Rechtschaffen & Kales, 1968	Determine troponin and CRP levels in people receiving haemodialysis with and without sleep apnoea
Kuhlmann et al., 2000 [33]	Germany	Haemodialysis	*n* = 77	55±17.2 years; *n* = 11/15 (78%)	Cross-sectional	Polygraphy; AHI ≥5	Not reported.	Not reported	Not reported	Determine prevalence of sleep disordered breathing
Lanis et al., 2018 [35]	USA	Peritoneal Dialysis	*n* = 15	51 (15) years; *n* = 10 (66%)	Cross-sectional	Polygraphy; AHI >5	Absence of airflow >10 seconds	Hypopnea with 4% desaturation	AASM 2012	Investigate sleep apnoea prevalence and to investigate the effect of peritoneal dialysis factors
Lyons et al., 2015 [48]	Canada	Haemodialysis	*n* = 28	53.5±10.4 years;	Before after (no control group)	Polysomnography; AHI ≥20	Central apnoea was defined as ≥90% reduction in tidal volume for ≥10 s in which thoracoabdominal motion was absent	Central hypopnea as a reduction of ≥30% in tidal volume for ≥10 s with in-phase thoracoabdominal motion without airflow limitation on nasal pressure accompanied by a ≥3% desaturation or an arousal	AASM 2012	Investigate the effect of ultrafiltration on sleep apnoea
Mahajan et al., 2018 [34]	India	Haemodialysis	*n* = 25	28.9±8.9 years (*n* = 18); *n* = 14/18 (78%)	Before after (no control group)	Polysomnography; AHI ≥5	Apnoea was defined as cessation of oro-nasal airflow for ≥ 10 seconds	Hypopnoea was defined as a discernible reduction in respiratory airflow during a preceding period of normal breathing for ≥10 sec accompanied by a decrease of 4% or more in oxyhaemoglobin saturation during sleep	Not reported.	Investigate the effect of kidney transplantation on sleep disordered breathing
Mendelson et al., 1990 [76]	USA	Haemodialysis	*n* = 11	53.8±2.5 years (SEM); *n* = 8/11 (73%)	Observational Cohort	Polysomnography; DBE (threshold not reported).	Absence of airflow >10 seconds, accompanied by a drop of at least 5% in arterial saturation.	Hypopneas comprised similar events in which air flow in the thermistor channels decreased to one-third the basal level	Rechtschaffen & Kales, 1968	Investigate the effect of haemodialysis treatment on sleep apnoea
Moradzadeh et al., 2021 [77]	Iran	Haemodialysis	*n* = 47	Low risk group: 58.54±14.32 years (*n* = 22); high risk group: 64.92±11.98 years (*n* = 25); *n* = 20/47 (43%)	Cross-sectional	Polygraphy; AHI ≥5	Not reported.	Not reported	Not reported	Investigate the prevalence and associated factors of sleep apnoea
Mucenica et al., 2006 (Conference Abstract) [22]	Romania	Haemodialysis	*n* = 19	49.6 years; 10/19 (53%)	Cross-sectional	Polysomnography; AHI >10	Not reported	Not reported	Not reported	Investigate the prevalence of sleep apnoea
Nicholl et al., 2012 [7]	Canada	Haemodialysis and Pre-Dialysis CKD	*n = *403; *n* = 75 (haemodialysis)	61.9±14.7 years; *n* = 52 (69%)	Cross-sectional	Polygraphy; RDI ≥15	Not reported	Not reported	AASM 2009	Investigate the prevalence of sleep apnoea
Ogna et al., 2015 [44]	Switzerland	Haemodialysis	*n* = 125; *n* = 104 (completers)	61.7±14.9 years; *n* = 66/104 (63%)	Cross-sectional	Polygraphy; AHI ≥15	Not reported	Not reported	AASM 2007	Investigate the prevalence of sleep apnoea
Parker et al., 2003 [39]	USA	Haemodialysis	*n* = 45	51.6±10.8 years; *n* = 24/46 (52%)	Cross-sectional	Polysomnography; RDI >5	A clear decrease (>50%) from baseline in the amplitude of a valid measure of breathing during sleeping; 2) or a clear amplitude reduction of a validated measure of breathing associated with either an oxygen desaturation of >3% or an arousal; 3) and the event lasts 10 s or longer	Hypopneas were not distinguished from apnoeas	Rechtschaffen & Kales, 1968	Establish the presence or absence of daytime sleepiness and associated variables
Pressman et al., 1993 [19]	USA	Haemodialysis, Peritoneal Dialysis and Pre-Dialysis CKD	n = 8	52±15 years; *n* = 6/8 (75%)	Before after (no control group)	Polysomnography; AHI ≥5	Defined by the complete absence of airflow with continued respiratory effort for at least 10 seconds with associated O_2_ desaturation ≥ 2% and/or clear electrographic signs of arousal or awakening from sleep	Hypopneas were defined by at least a 20% reduction in airflow and/or effort lasting for at least 10 seconds	Rechtschaffen & Kales, 1968	Investigate the of treating sleep apnoea with CPAP
Rodrigues et al., 2010 [37]	Brazil	Haemodialysis	*n* = 46; *n* = 34 completers	35±10.4 years (*n* = 34); *n* = 20/34 (58%)	Before after (no control group)	Polysomnography; AHI ≥5	1) A clear decrease (>50%) from baseline in the amplitude of a valid measure of breathing during sleeping; 2) or a clear amplitude reduction of a validated measure of breathing during sleep that does not reach the above criterion but is associated with either an oxygen desaturation of >3% or an arousal; 3) and the event lasts 10 s or longer	Hypopneas were not distinguished from apnoeas	AASM 1999	Investigate the effect of kidney transplantation on sleep disordered breathing
Rodriguez et al., 1995 [38]	USA	Peritoneal Dialysis	*n = *20	58±12 years (*n* = 18); *n* = 9/18 (50%)	Cross-sectional	Polygraphy; RDI ≥5	Not reported	Not reported	Not reported	Investigate the prevalence of sleep apnoea
Roumelioti et al., 2011 [26]	USA	Haemodialysis and Normal Kidney Function Controls	*n* = 388; *n* = 75 (haemodialysis)	57.5 (46, 67.2) years (*n* = 75); *n* = 49/75 (66.2%)	Cross-sectional	Polysomnography; AHI ≥30	Apnoea was defined as a complete or an almost complete (≥25% of baseline) airflow cessation; measured by the amplitude of the ≥10-second nasal pressure signal	Hypopnea was defined as a ≥10 second abnormal respirator event with ≥30% airflow reduction (compared with baseline) and was associated with ≥4% oxyhemoglobin desaturation	Rechtschaffen & Kales, 1968	Investigate the prevalence of sleep disordered breathing and associated factors
Roumelioti et al., 2016 [27]	USA	Peritoneal dialysis	*n* = 186; *n* = 22 (peritoneal dialysis)	37.5 (31.5, 58.3) years (*n* = 22); *n* = 11/22 (50%).	Cross-sectional	Polysomnography; AHI ≥15	Apnoea was defined as a complete or an almost complete (≥25% of baseline) airflow cessation; measured by the amplitude of the ≥10-second nasal pressure signal.	Hypopnea was defined as a ≥10 second abnormal respiratory event with ≥30% airflow reduction (compared with baseline) and was associated with ≥4% oxyhemoglobin desaturation.	Rechtschaffen & Kales, 1968	Investigate the prevalence of sleep disordered beathing
Sakura et al., 2016 [28]	Japan	Haemodialysis	*n* = 517; *n* = 73 (haemodialysis)	68.8±9.0 years (*n* = 73); *n* = 46/73 (63%)	Observational cohort	Polysomnography; AHI ≥ 5	Apnoea was defined as a cessation of airflow for at least 10 s	Hypopnea was defined as a ≥ 50% decrease in the amplitude, resulting in a decrease of at least 3% in the arterial oxyhemoglobin saturation	AASM 2007	Investigate sleep apnoea in people receiving haemodialysis
Sanner et al., 2002 [40]	Germany	Haemodialysis	*n* = 33	66 (22-82) years; Sex not reported	Cross-sectional	Polygraphy; AHI ≥5	A complete cessation of airflow lasting ≥10 s	A reduction in respiratory airflow of ≥50% of the airlfow lasting >10 s associated with a desaturation of >3% could be discerened (hyponea)	AASM 2009	Investigate the effect of sleep disordered breathing on quality of life
Sivalingam et al., et al., 2013 [41]	United Kingdom	Haemodialysis	*n* = 102	Major obstructive sleep apnoea (MOSA) group = 59.0±12.0 years (*n* = 40); No MOSA group = 61.4±16.6 years (*n* = 50); MOSA group = 34/40 (86%); No MOSA group = 25/50 (50%)	Observational cohort	Polygraphy; AHI >5	Apnoea was defined as complete cessation of airflow	Hypopnea as a detectable reduction in airflow or thoracoabdominal excursion of at least 50% from baseline, lasting 10 seconds or more	Not reported	Investigate the prevalence and determinants of sleep disturbances
Tang et al., 2012 (Conference Abstract) [23]	USA	Peritoneal dialysis	*n* = 17	47.7±16.9. years; *n* = 14/17 (82%)	Before after (no control group)	Polysomnography; AHI >5	Not reported.	Not reported.	Not reported.	Investigate the effect of kidney transplantation on sleep apnoea.
Tang et al., 2006 [42]	USA	Peritoneal dialysis	*n* = 46	Nocturnal PD = 61.4±16.8; Continuous Ambulatory PD = 60.2±16.7; Nocturnal PD = 11/23 (47%); Continuous Ambulatory PD = 12/23 (53%)	Cross-sectional	Polysomnography; AHI ≥15	Apnoea was defined as the cessation of airflow for >10 s	Defined as a reduction of airflow of ≥50% for >10 s plus an oxygen desaturation of ≥4%	AASM 1992	Investigate the prevalence of sleep apnoea and the effect of peritoneal dialysis treatments
Venmans et al., 1999 [43]	Netherlands	Haemodialysis	*n* = 16	50 (23-75) years (*n* = 15); *n* = 9/15 (60%).	Observational cohort	Polygraphy; AHI>5	Defined as a cessation in airflow lasting at least 10 s	A hypopnea was defined as a 50% reduction in airflow, lasting at least 10 s, and associated with a 4% or greater decrease in saturation	Not reported	To investigate the prevalence of sleep disordered breathing

Data reported as either mean ± SD or median (IQR) unless otherwise stated. American Academy of Sleep Medicine (AASM), Apnoea-Hypopnea Index (AHI), Disordered Breathing Events (DBE), Respiratory Disturbance Index (RD), s (seconds)

### Characteristics of included studies

Of the 36 separate studies there were nineteen cross-sectional, eight observational cohort, seven before-after (no control group), and two controlled interventional studies (Table 1). Three of these studies [21–23] were reported as conference abstracts. There were 1,399 included participants and we included only the individuals receiving dialysis from six studies which also reported data for other CKD and healthy control populations [7, 24–28]. Twenty-two studies included people receiving haemodialysis, six included people receiving peritoneal dialysis, three studies contained people receiving both types of dialysis, and five contained subjects on dialysis alongside other populations (Table 1). Sleep apnoea was classified using the apnoea-hypopnea index in thirty studies, four studies used the respiratory disturbance index, and two used disordered breathing events, respectively (Table 1). For the apnoea-hypopnea index, the threshold to classify sleep disordered breathing ranged from > 5 to ≥ 30, for respiratory disturbance index, the threshold ranged from > 5 to ≥ 15, whilst for disordered breathing events, the threshold was 5 per hour (5/h) (Table 1).

## Quality rating for the included studies

### Observational cohort and cross sectional studies

**Fig. 2:**
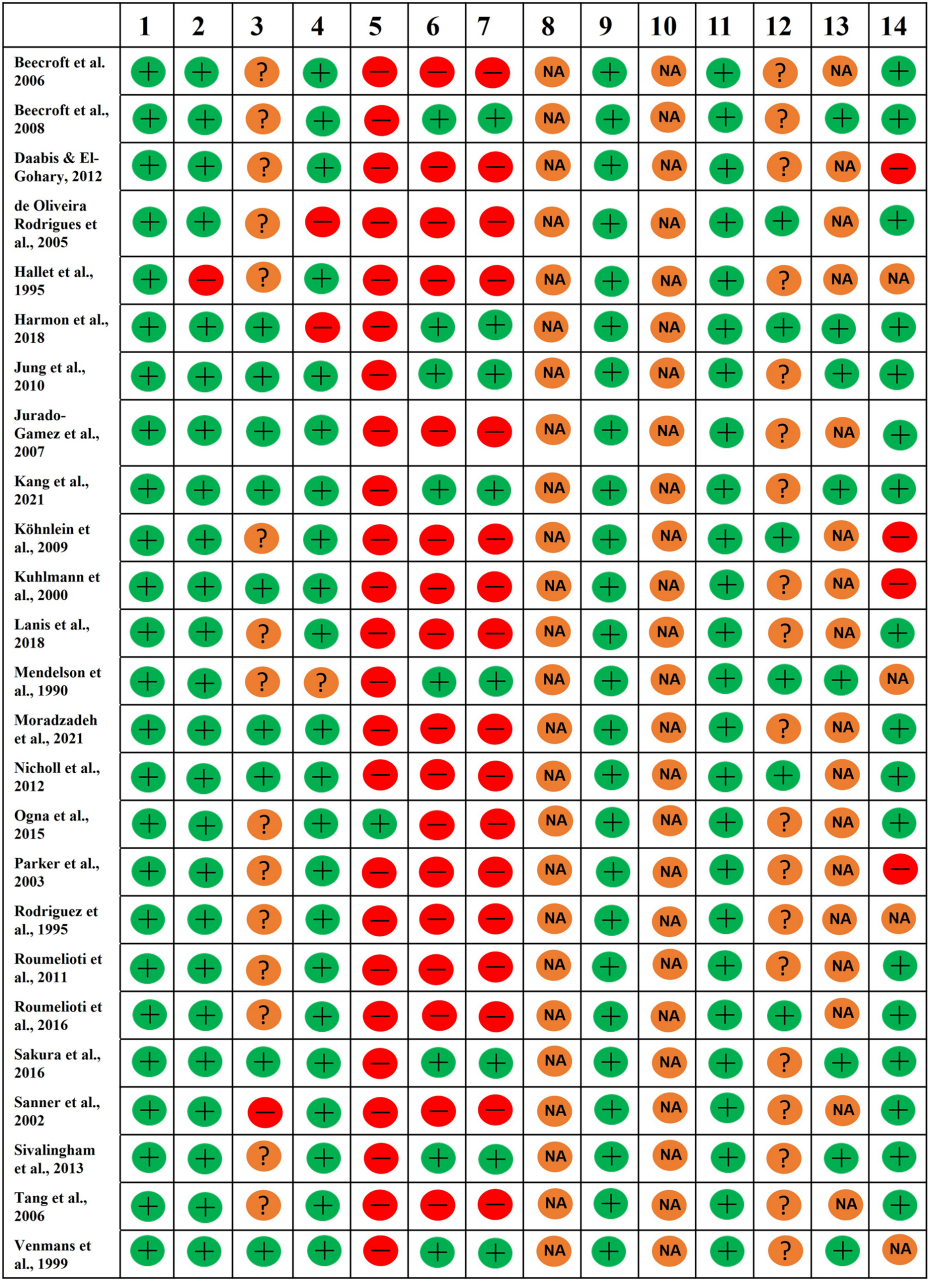
Quality assessment of included cohort and cross-sectional studies. As all participants were individuals with kidney failure (which was defined as the exposure), and this does not vary in amount or level, Question 8 was thus classified as not applicable (NA). This exposure was not assessed more than once over time, so Question 10 was classified as NA. Sleep apnoea was defined as the outcome

Only one study (*n* = 1/25) reported a sample size calculation (Question 5). Most studies (*n* = 17/25) were limited by the nature of cross-sectional design (Questions 6 & 7) (Fig. 2), and only six (*n* = 6/25) reported blinding of outcome assessors (Question 12) (Fig. 2).

### Before-after (pre-post) and controlled intervention studies

**Fig. 3:**
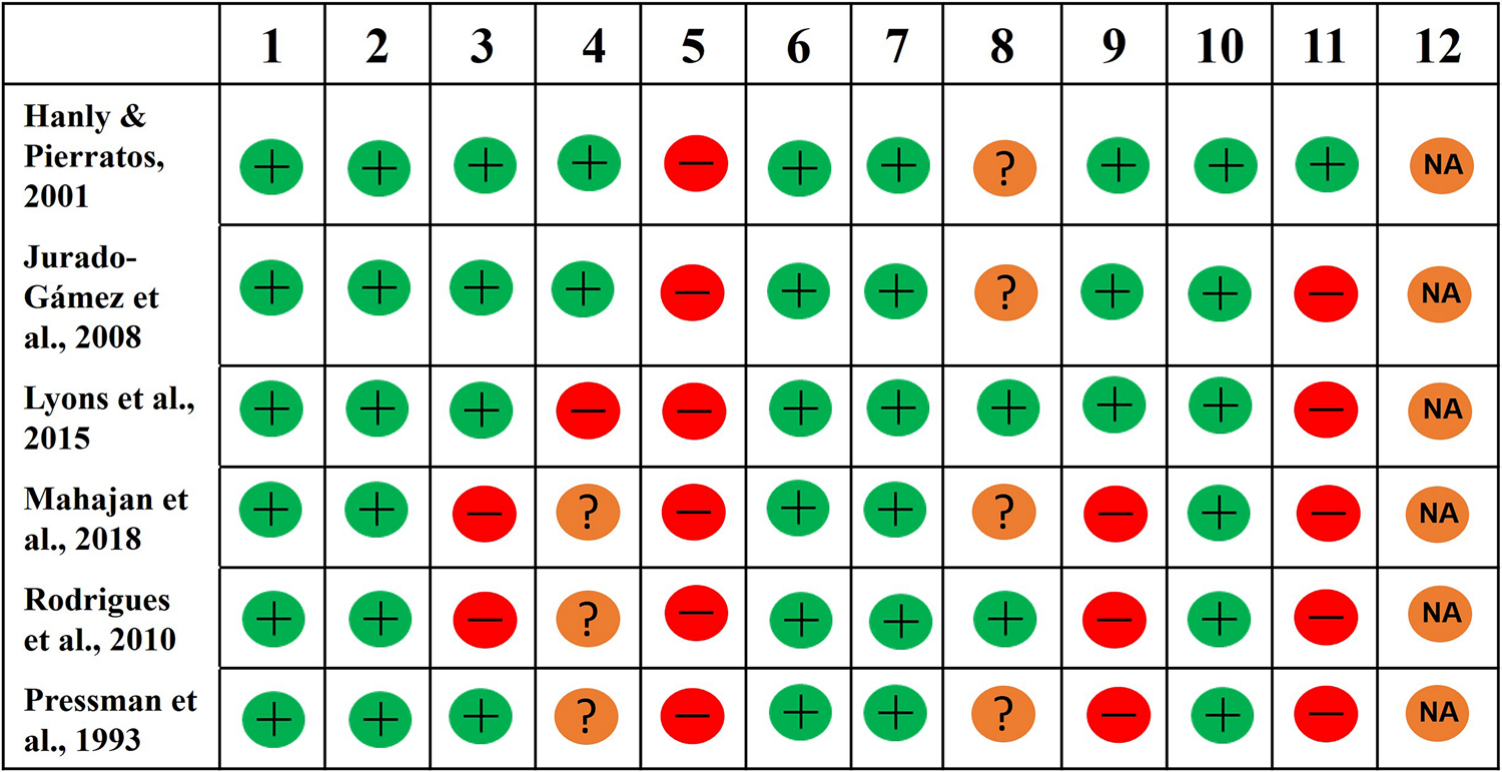
Quality assessment of included before-after (pre-post) studies with no control group. Question 12 was classified as not applicable as interventions were applied at the participant level

**Fig. 4:**
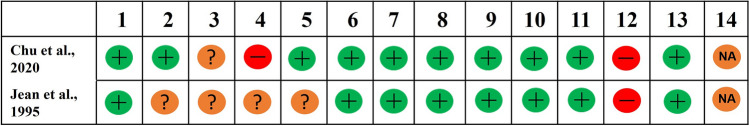
Quality assessment of included controlled intervention studies. Question 14 was classified as not applicable as both studies were randomised crossover studies

For the before-after (pre-post) studies, none were sufficiently large (*n* ≥ 50) (Question 5) (Fig. 3), and only *n* = 1/6 reported taking outcome measures multiple times before and after the intervention (Question 11) (Fig. 3). For the two controlled intervention studies there were deficiencies for treatment allocation concealment (Question 3), blinding (Question 4), and sample size (n ≥ 50) (Question 12) (Fig. 4).

### Prevalence of sleep apnoea in the dialysis population

**Fig. 5:**
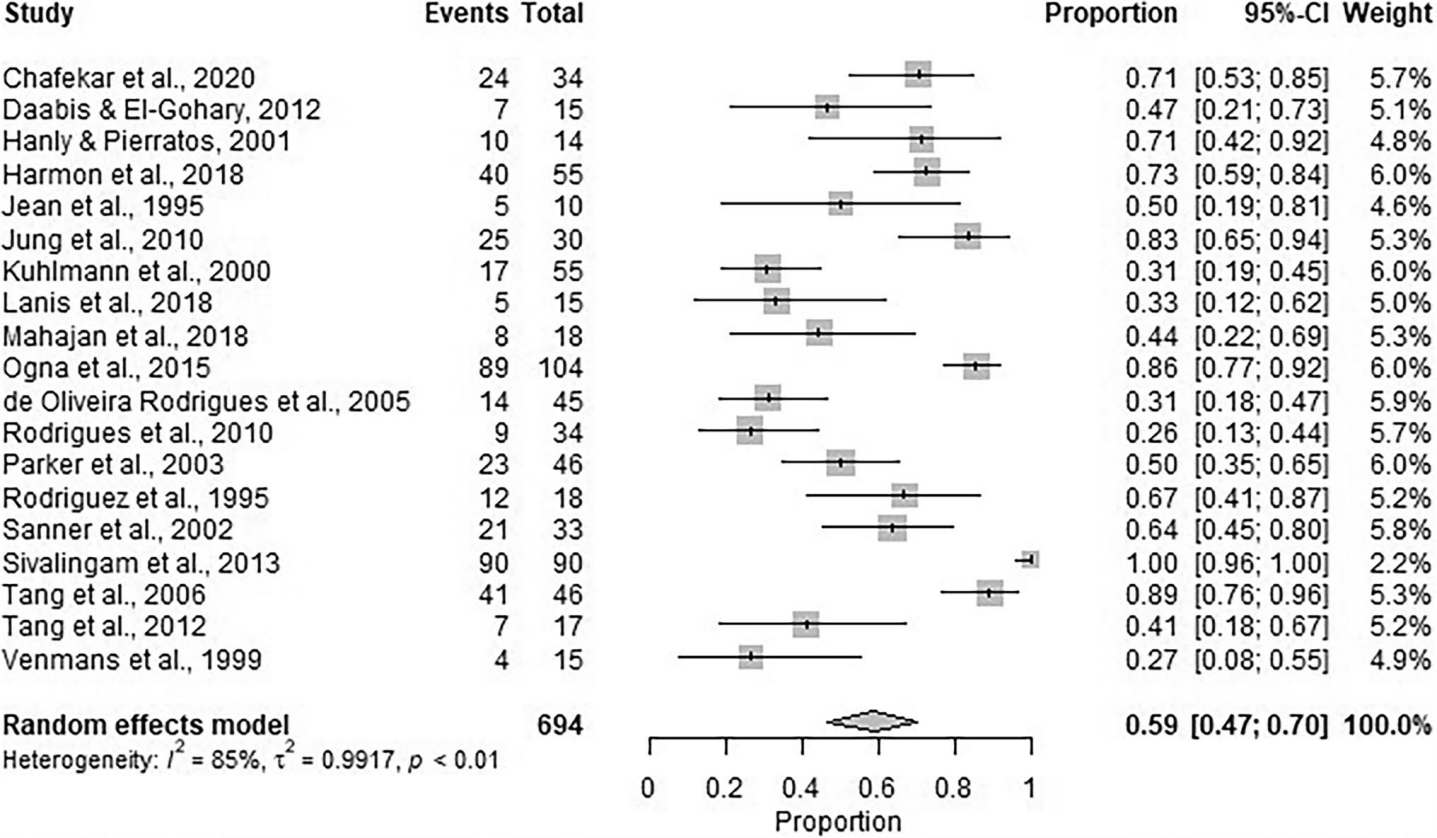
Pooled proportion of participants with apnoea across studies. Apnoea defined as Apnoea-Hypopnea Index (AHI) > 5, AHI ≥ 5, respiratory disturbance index > 5 or disordered breathing events ≥ 5. Between-study heterogeneity (${\tau }^{2}$ is estimated to be 0.99 and the ${I}^{2}$ suggests that 85% of the total variability in the estimates is due to the heterogeneity

**Fig. 6:**
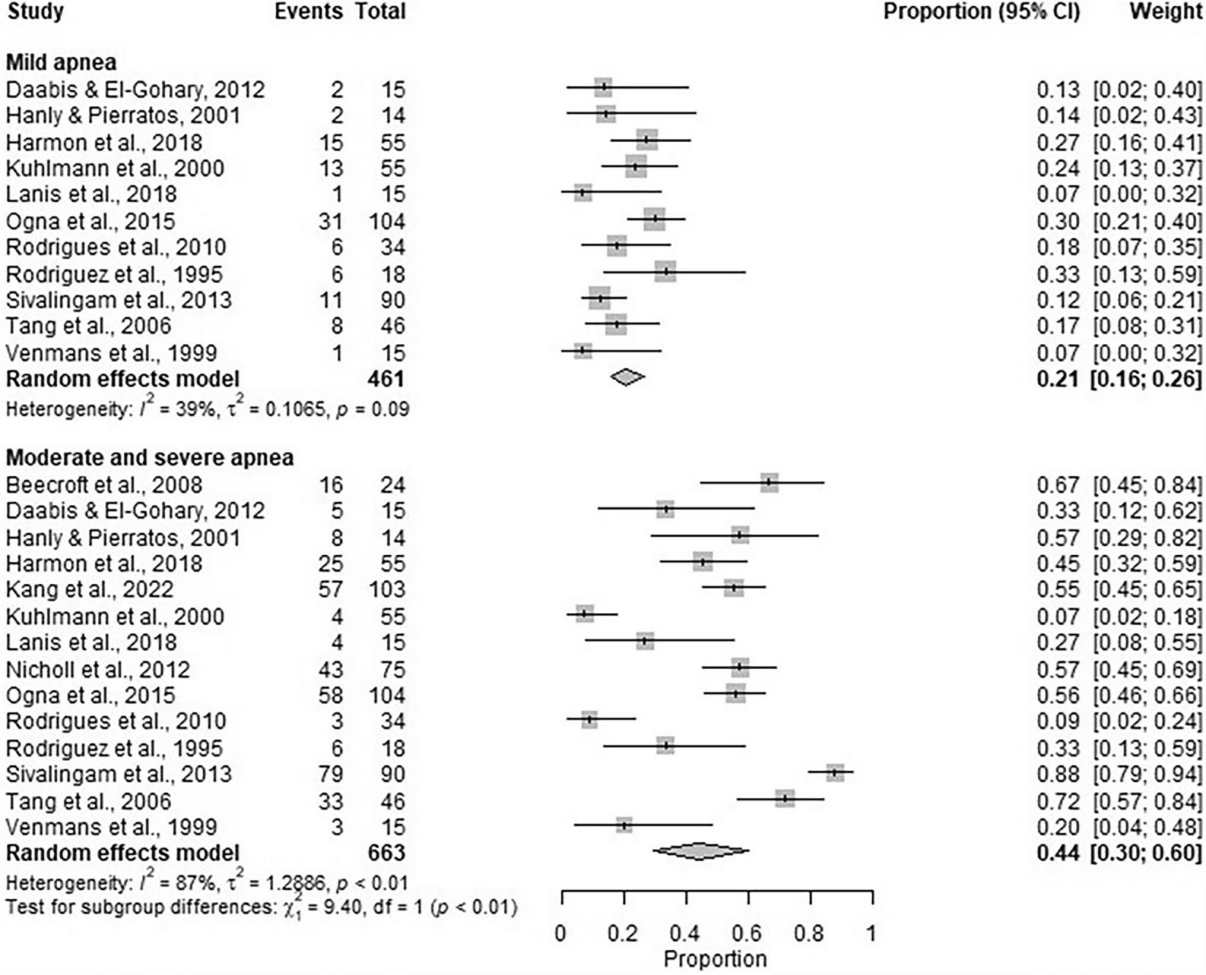
Pooled proportion of participants with mild (Apnoea-Hypopnea Index [AHI] > 5 or AHI ≥ 5 to AHI 14), moderate and severe (AHI ≥ 15 or respiratory disturbance index ≥ 15) sleep apnoea. The between-study heterogeneity is higher in the moderate and severe subgroup, estimated as 1.29 (${I}^{2}$=87%), compared to the mild sleep apnoea subgroup where between-study heterogeneity is estimated to be 0.11 (${I}^{2}$=39%). The chi-squared test for subgroup difference suggests a significant difference between the proportion of participants with mild and moderate and severe sleep apnoea in the studies (*p* < 0.01)

Data from 19 studies [21, 23, 24, 29–44] reporting sleep apnoea (apnoea-hypopnea index > 5, apnoea-hypopnea index ≥ 5 or respiratory disturbance index > 5) were synthesised. The pooled proportion of participants that had sleep apnoea across studies was estimated to be 59% (95% CI: 47%, 70%) (Fig. 5). The number of studies with data suitable for synthesis that reported the proportion of participants classified with mild apnoea (apnoea-hypopnea index > 5 or apnoea-hypopnea index ≥ 5 to apnoea-hypopnea index < 15) was 11 [24, 29, 30, 33, 35, 37, 38, 41–44], and 14 for moderate and severe (apnoea-hypopnea index ≥ 15 or respiratory disturbance index ≥ 15) sleep apnoea [7, 24, 29, 30, 33, 35, 37, 38, 41–46]. The pooled proportion of participants with mild sleep apnoea was estimated to be 21% (95% CI: 16%, 26%) (Fig. 6). The pooled proportion of participants with moderate and severe apnoea was estimated to be 44% (95% CI: 30%, 60%) (Fig. 6).

### Type of sleep apnoea in the dialysis population

**Fig. 7:**
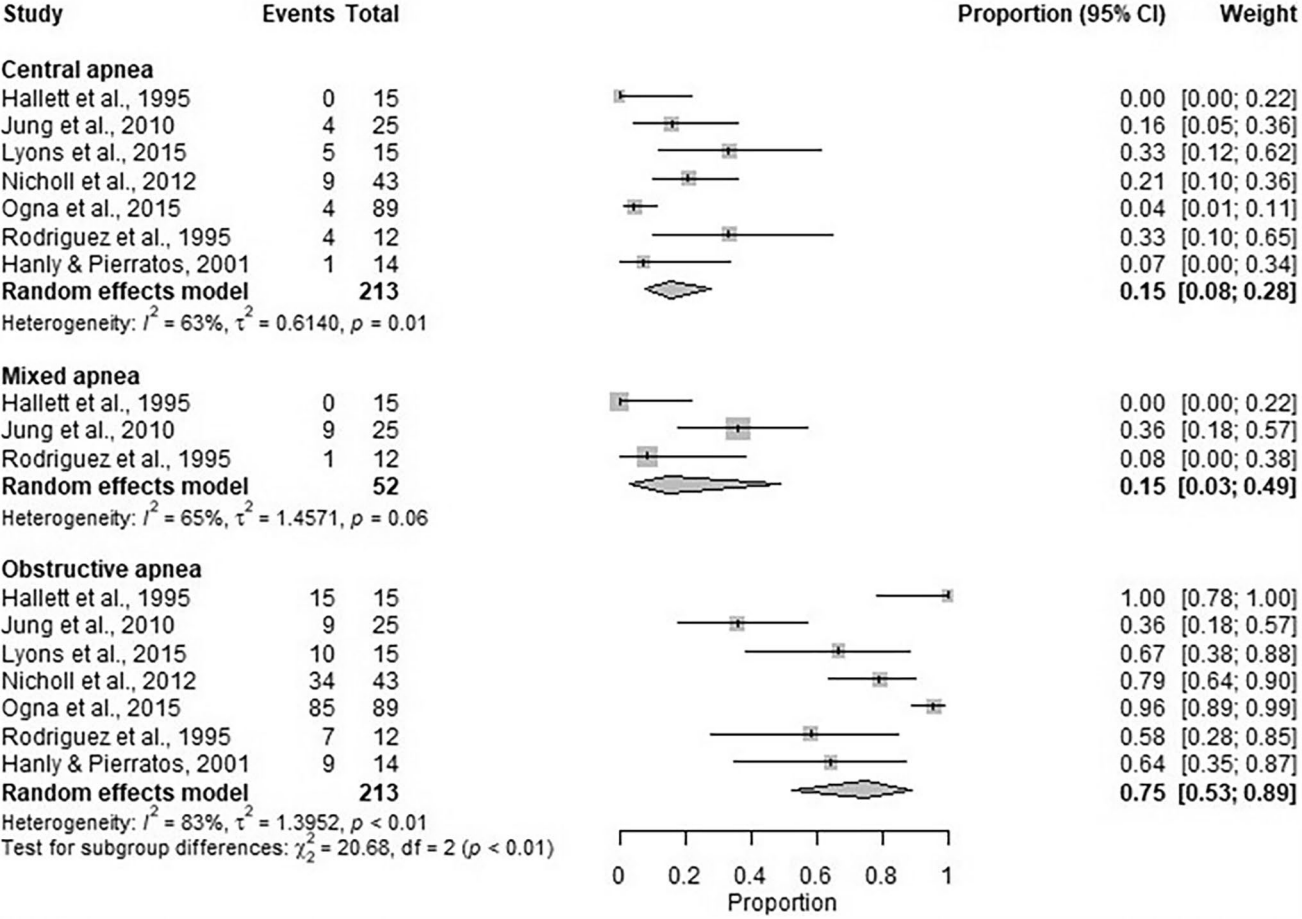
Pooled proportion of participants with obstructive, central and mixed sleep apnoea. The between-study heterogeneity is highest in the obstructive sleep apnoea subgroup, estimated as 1.40 (${I}^{2}$=83%), compared to the central sleep apnoea subgroup where between-study heterogeneity is estimated to be 0.61 (${I}^{2}$=63%), and mixed sleep apnoea subgroup where the between-study heterogeneity is estimated to be 1.46 (${I}^{2}$=65%). The chi-squared test for subgroup difference suggests a significant difference between the proportion of participants with the different types of sleep apnoea in the studies (*p* < 0.01)

There were seven studies [7, 29, 32, 38, 44, 47, 48] which reported data for type of sleep apnoea. Pooled proportion of participants with apnoea which was obstructive was estimated to be 75% (95% CI: 53%, 89%), central was estimated to be 15% (95% CI: 8%, 28%), with mixed estimated also at 15% (95% CI: 3%, 49%) (Fig. 7).

### Interventions for sleep disordered breathing in the dialysis population

One study [29] involving fourteen participants investigated the effect of changing haemodialysis treatment from conventional thrice weekly to extended overnight treatment. They reported that the change in dialysis regimen resulted in a reduction in sleep apnoea [29]. Two further randomised studies [20, 31] investigated the effect of haemodiafiltration compared to conventional haemodialysis [20], and the effect of both acetate and bicarbonate dialysate buffer [31] on sleep apnoea. There was no difference reported between haemodiafiltration compared to conventional haemodialysis on apnoea-hypopnea index [20]. The second randomised study [31] reported more frequent sleep apnoea following haemodialysis treatment with acetate buffer compared to dialysate buffer. A study including 46 people receiving peritoneal dialysis found that levels of sleep apnoea were lower in a group of people receiving nocturnal cycler-assisted peritoneal dialysis compared to conventional peritoneal dialysis treatment [42]. A further study has reported that ultrafiltration may reduce apnoea-hypopnea index events [48]; they concluded that fluid overload may play a key role in sleep disturbance. Four observational cohort studies investigated the effect of kidney transplantation on sleep apnoea [23, 34, 37, 49]. Three reported a decrease in apnoea-hypopnea index following transplantation [23, 34, 49], with one [37] reporting no change. Lastly, we found one study that had investigated the effect of acute CPAP in people receiving dialysis [19]. They reported a significant decrease in apnoea-hypopnea index following CPAP treatment [19].

## Discussion

This is the first review which has reported on the prevalence of mild and moderate sleep apnoea in people with kidney failure receiving dialysis. The main findings of the review were that overall prevalence of sleep apnoea was 59%, with prevalence of mild apnoea estimated to be 21%, and moderate and severe prevalence estimated at 44%. Sleep apnoea was predominantly obstructive in nature, with prevalence estimated at 75% for obstructive, and 15% for both central and mixed based on the available data. There was limited evidence for effective interventions for sleep apnoea, which included changes to dialysis treatment and kidney transplantation; only one included study assessed the effect of CPAP.

A recent systematic review [13] reported that prevalence of sleep apnoea was 56% (95% CI: 0.42%, 0.69%) in kidney failure patients, which was assessed by using instrumental monitoring devices. When the analysis investigated prevalence reported from studies that had used sleep questionnaires it was estimated to be lower, at 39% (95% CI: 30%, 49%) [13]. These data support a previous study showing that screening questionnaires do not accurately identify obstructive sleep apnoea in the kidney failure population [50]. However, it is worth highlighting that in a recent review [13], the data from the individual studies (included in the pooled analysis) had been based on different criteria (e.g. mild ( apnoea-hypopnea index ≥ 5), moderate ( apnoea-hypopnea index ≥ 15), severe apnoea-hypopnea index ≥ 30) to define sleep apnoea, and included studies [9, 19, 51–54] where the population were not all receiving haemodialysis and had been pre-screened [55–57] for sleep-disordered breathing. Despite this, reassuringly, they have reported a similar prevalence to the data we present in this review. When they [13] performed a subgroup analysis including studies only with an apnoea-hypopnea index/respiratory disturbance index threshold of ≥ 15, they reported a prevalence of 56% (95% CI: 38%, 75%), which is higher than the value we report of 44% (95% CI: 30%, 60%). This may be explained by the aforementioned differences in the included studies between reviews. Another meta-analysis reported the prevalence of sleep apnoea to be 35.1% (95% CI: 25.8%, 45.8%) in dialysis-dependent individuals, however they included studies that used questionnaires to define obstructive sleep apnoea [58]. We found that three [23, 34, 49] of our included studies reported an improvement in sleep apnoea following kidney transplantation. However, a recent meta-analysis (which included two of these three studies) comparing sleep apnoea before and after kidney transplantation reported no reduction in apnoea-hypopnea index when the data were pooled [59].

Our review demonstrated a high prevalence of sleep apnoea, with the majority appearing to be moderate and severe in nature. Based on this review, there may be value in screening people receiving dialysis for the presence of sleep apnoea (particularly as there is a likelihood that this could be underdiagnosed) with the view to refer them to sleep disordered services. We acknowledge that there is insufficient evidence that this approach would improve outcomes or would be cost-effective. Questionnaires such as the STOP-Bang (which is recommended by the National Institute for Health and Care Excellence in the UK for the general population) are believed to underestimate prevalence of sleep apnoea [13, 50, 60] and therefore may not be suitable for pre-screening, while other methods such as oximetry may be more suitable. Alongside the findings of this review, we highlight the need to collect further evidence for the management and treatment of sleep apnoea in kidney failure. This is further emphasised by the fact that although sleep disturbances are recognised as a common symptom of CKD within clinical practice guidelines [61], there are no recommendations for the management of such symptoms [61, 62].

There are several unique factors that may contribute to the association between kidney failure and sleep apnoea. These include changes to chemoreflex responsiveness [63] as a result of the uraemic environment, fluid overload [64], anaemia [65], and inflammation [25]. However, as previously discussed, this review did not identify sufficient evidence for the effective treatment of sleep apnoea. In both the general population and for those receiving dialysis treatment, CPAP is the first line of treatment for people with mild to moderate sleep apnoea. However, we only identified one study [19] that had tested this treatment in people receiving dialysis. It reported some evidence of effectiveness following acute treatment with CPAP, although this study [19] was small and non-randomised. Individuals receiving dialysis have an elevated cardiovascular risk, and sleep apnoea my contribute to this risk [66], therefore it is reasonable to assume that CPAP may improve outcome in the kidney failure population. However, a previous trial [67] in individuals with obstructive sleep apnoea and established coronary or cerebrovascular disease did not show any benefit of CPAP on their cardiovascular composite end point. Moreover, somewhat surprisingly, a further trial [68] showed that adaptive servo-ventilation therapy for people with a reduced ejection fraction and central sleep apnoea increased all-cause and cardiovascular mortality. Given the increased cardiovascular risk in individuals with kidney failure (and the presence of central sleep apnoea), it is imperative that future randomised trials investigate the efficacy and safety of CPAP for the treatment of individuals with apnoea receiving dialysis.

The body of literature identified by this scoping review was sufficient to estimate the prevalence of sleep apnoea in this population, however we did not identify sufficient volume to recommend effective treatments. The evidence presented in this review is limited by the design of the studies (being of cross-sectional nature), although we excluded studies that had preselected individuals (based on history of sleep disordered breathing or selection via questionnaires or oximetry) in order to reduce bias for the prevalence data reported. Most studies were not powered, and lack of blinding for assessing sleep apnoea was a common theme identified in the quality assessment. There was a large range (from apnoea-hypopnea index > 5 to apnoea-hypopnea index ≥ 30) in the included studies with regard to the threshold used to define sleep apnoea. We have attempted to account for this in our meta-analysis.

## Conclusion

In conclusion, there is a high prevalence of sleep apnoea in the dialysis population, with most of this being obstructive in nature. Currently, there is insufficient evidence for the treatment of sleep apnoea in this patient population. Appropriately powered randomised controlled trials are required to investigate the efficacy and safety of treatments for apnoea in the dialysis population.

## Supplementary Information

Below is the link to the electronic supplementary material.

Supplementary file1 (DOCX 39 KB)

## Data Availability

The data that support the findings of this study (from the three meta-analyses) are publicly available at: https://figshare.com/articles/dataset/Meta-analysis_raw_data_Prevalence_and_characteristics_of_treatments_for_sleep_disordered_breathing_in_people_receiving_dialysis_A_Scoping_Review/26893147?file=48932722.
