# Supplementary material for: Prevalence and characteristics of treatments for sleep disordered breathing in people receiving dialysis: a scoping review
Source: J Nephrol. 2025 Sep 9;38(8):2129–54. doi: 10.1007/s40620-025-02370-x (PMC12630318; doi:10.1007/s40620-025-02370-x)
Supplement: Supplementary file 1 — Supplementary file1 (DOCX 39 KB) [file 40620_2025_2370_MOESM1_ESM.docx]

**S1 Table. Preferred reporting items for systematic reviews and meta-analyses extension for scoping reviews (PRISMA-ScR) checklist**

| **SECTION** | **ITEM** | **PRISMA-ScR CHECKLIST ITEM** | **REPORTED ON PAGE #** |
| --- | --- | --- | --- |
| **TITLE** | | | |
| Title | 1 | Identify the report as a scoping review. | 1 |
| **ABSTRACT** | | | |
| Structured summary | 2 | Provide a structured summary that includes (as applicable): background, objectives, eligibility criteria, sources of evidence, charting methods, results, and conclusions that relate to the review questions and objectives. | 1&2 |
| **INTRODUCTION** | | | |
| Rationale | 3 | Describe the rationale for the review in the context of what is already known. Explain why the review questions/objectives lend themselves to a scoping review approach. | 2&3 |
| Objectives | 4 | Provide an explicit statement of the questions and objectives being addressed with reference to their key elements (e.g., population or participants, concepts, and context) or other relevant key elements used to conceptualize the review questions and/or objectives. | 3 |
| **METHODS** | | | |
| Protocol and registration | 5 | Indicate whether a review protocol exists; state if and where it can be accessed (e.g., a Web address); and if available, provide registration information, including the registration number. | 3&4 |
| Eligibility criteria | 6 | Specify characteristics of the sources of evidence used as eligibility criteria (e.g., years considered, language, and publication status), and provide a rationale. | 4&5 |
| Information sources* | 7 | Describe all information sources in the search (e.g., databases with dates of coverage and contact with authors to identify additional sources), as well as the date the most recent search was executed. | 4&5 |
| Search | 8 | Present the full electronic search strategy for at least 1 database, including any limits used, such that it could be repeated. | 3&4 (Supplementary Material) |
| Selection of sources of evidence† | 9 | State the process for selecting sources of evidence (i.e., screening and eligibility) included in the scoping review. | 4 |
| Data charting process‡ | 10 | Describe the methods of charting data from the included sources of evidence (e.g., calibrated forms or forms that have been tested by the team before their use, and whether data charting was done independently or in duplicate) and any processes for obtaining and confirming data from investigators. | 4 |
| Data items | 11 | List and define all variables for which data were sought and any assumptions and simplifications made. | 5 |
| Critical appraisal of individual sources of evidence§ | 12 | If done, provide a rationale for conducting a critical appraisal of included sources of evidence; describe the methods used and how this information was used in any data synthesis (if appropriate). | 5 |
| Synthesis of results | 13 | Describe the methods of handling and summarizing the data that were charted. | 4&5 |
| **RESULTS** | | | |
| Selection of sources of evidence | 14 | Give numbers of sources of evidence screened, assessed for eligibility, and included in the review, with reasons for exclusions at each stage, ideally using a flow diagram. | Figure 1 |
| Characteristics of sources of evidence | 15 | For each source of evidence, present characteristics for which data were charted and provide the citations. | 17, 18, 19; Table 1 |
| Critical appraisal within sources of evidence | 16 | If done, present data on critical appraisal of included sources of evidence (see item 12). | 17; Figures 2, 3 & 4 |
| Results of individual sources of evidence | 17 | For each included source of evidence, present the relevant data that were charted that relate to the review questions and objectives. | 18&19; Table 1 |
| Synthesis of results | 18 | Summarize and/or present the charting results as they relate to the review questions and objectives. | 18&19, Figures 5, 6 & 7 |
| **DISCUSSION** | | | |
| Summary of evidence | 19 | Summarize the main results (including an overview of concepts, themes, and types of evidence available), link to the review questions and objectives, and consider the relevance to key groups. | 19, 20 & 21 |
| Limitations | 20 | Discuss the limitations of the scoping review process. | 21 |
| Conclusions | 21 | Provide a general interpretation of the results with respect to the review questions and objectives, as well as potential implications and/or next steps. | 21 |
| **FUNDING** | | | |
| Funding | 22 | Describe sources of funding for the included sources of evidence, as well as sources of funding for the scoping review. Describe the role of the funders of the scoping review. | 22 |

JBI = Joanna Briggs Institute; PRISMA-ScR = Preferred Reporting Items for Systematic reviews and Meta-Analyses extension for Scoping Reviews.

* Where *sources of evidence* (see second footnote) are compiled from, such as bibliographic databases, social media platforms, and Web sites.

† A more inclusive/heterogeneous term used to account for the different types of evidence or data sources (e.g., quantitative and/or qualitative research, expert opinion, and policy documents) that may be eligible in a scoping review as opposed to only studies. This is not to be confused with *information sources* (see first footnote).

‡ The frameworks by Arksey and O’Malley (6) and Levac and colleagues (7) and the JBI guidance (4, 5) refer to the process of data extraction in a scoping review as data charting*.*

§ The process of systematically examining research evidence to assess its validity, results, and relevance before using it to inform a decision. This term is used for items 12 and 19 instead of "risk of bias" (which is more applicable to systematic reviews of interventions) to include and acknowledge the various sources of evidence that may be used in a scoping review (e.g., quantitative and/or qualitative research, expert opinion, and policy document).

*From:* Tricco AC, Lillie E, Zarin W, O'Brien KK, Colquhoun H, Levac D, et al. PRISMA Extension for Scoping Reviews (PRISMAScR): Checklist and Explanation. Ann Intern Med. 2018;169:467–473. [doi: 10.7326/M18-0850](http://annals.org/aim/fullarticle/2700389/prisma-extension-scoping-reviews-prisma-scr-checklist-explanation).

| **S2 Table. Full Search Strategy for MEDLINE** | | |
| --- | --- | --- |
|  | **Search Item** | **Field** |
|  | Renal insufficiency | MH explode |
|  | Renal insufficienc* | MP |
|  | Renal insufficiency, chronic | MH explode |
|  | Chronic kidney insufficiency | MP |
|  | Chronic kidney disease* | MP |
|  | Chronic renal disease* | MP |
|  | Kidney failure, chronic | MH explode |
|  | Chronic kidney failure | MP |
|  | End Stage Kidney Disease | MP |
|  | End Stage Renal Disease | MP |
|  | End Stage Renal Failure | MP |
|  | Chronic Renal Failure | MP |
|  | Renal Replacement Therapy | MH explode |
|  | Renal Replacement Therapy | MP |
|  | ESKD | MP |
|  | ESRD | MP |
|  | Renal dialysis | MH explode |
|  | Renal dialysis | MP |
|  | Hemodialysis | MP |
|  | Haemodialysis | MP |
|  | Extracorporeal Dialysis | MP |
|  | Peritoneal dialysis | MH explode |
|  | Peritoneal dialysis | MP |
|  | Peritoneal dialysis, continuous ambulatory | MH explode |
|  | Continuous Ambulatory peritoneal dialysis | MP |
|  | CAPD | MP |
|  | Renal replacement therapy | MH explode |
|  | Renal replacement Therap* | MP |
|  | Kidney Replacement Therapy | MP |
|  | Kidney transplantation | MH explode |
|  | Kidney transplantation* | MP |
|  | Renal Transplantation* | MP |
|  | Kidney Grafting | MP |
|  | Transplants | MH explode |
|  | Transplants | MP |
|  | Sleep apnea syndromes | MH explode |
|  | Sleep apn$ea syndrome* | MP |
|  | Sleep hypopn$ea* | MP |
|  | Sleep Apn$ea* | MP |
|  | Mixed Sleep Apn$ea* | MP |
|  | Hypersomnia with Periodic Respiration | MP |
|  | Sleep Disordered Breathing | MP |
|  | SDB | MP |
|  | Sleep apnea, central | MH explode |
|  | Central sleep apn$ea* | MP |
|  | Central Apn$ea* | MP |
|  | Central Sleep Apn$ea Syndrome | MP |
|  | Central Sleep Disordered Breathing | MP |
|  | Sleep apnea, obstructive | MH explode |
|  | Obstructive sleep apn$ea * | MP |
|  | Obstructive Sleep Apn$ea Syndrome | MP |
|  | OSAHS | MP |
|  | Sleep Apn$ea Hypopn$ea Syndrome | MP |
|  | Upper Airway Resistance Sleep Apn$ea Syndrome | MP |
| MH: MeSH terms; MP: keywords | | |

| **S3 Table. Criteria used to assess the quality of the included studies** | |
| --- | --- |
| **Controlled Intervention Study** | |
| **Number** | **Criteria** |
| 1 | Was the study described as randomized, a randomized trial, a randomized clinical trial, or an RCT? |
| 2 | Was the method of randomization adequate (i.e., use of randomly generated assignment)? |
| 3 | Was the treatment allocation concealed (so that assignments could not be predicted)? |
| 4 | Were study participants and providers blinded to treatment group assignment? |
| 5 | Were the people assessing the outcomes blinded to the participants' group assignments? |
| 6 | Were the groups similar at baseline on important characteristics that could affect outcomes (e.g., demographics, risk factors, co-morbid conditions)? |
| 7 | Was the overall drop-out rate from the study at endpoint 20% or lower of the number allocated to treatment? |
| 8 | Was the differential drop-out rate (between treatment groups) at endpoint 15 percentage points or lower? |
| 9 | Was there high adherence to the intervention protocols for each treatment group? |
| 10 | Were other interventions avoided or similar in the groups (e.g., similar background treatments)? |
| 11 | Were outcomes assessed using valid and reliable measures, implemented consistently across all study participants? |
| 12 | Did the authors report that the sample size was sufficiently large to be able to detect a difference in the main outcome between groups with at least 80% power? |
| 13 | Were outcomes reported or subgroups analyzed prespecified (i.e., identified before analyses were conducted)? |
| 14 | Were all randomized participants analyzed in the group to which they were originally assigned, i.e., did they use an intention-to-treat analysis? |
| **Observational Cohort and Cross-Sectional Study** | |
| **Number** | **Criteria** |
| 1 | Was the research question or objective in this paper clearly stated? |
| 2 | Was the study population clearly specified and defined? |
| 3 | Was the participation rate of eligible persons at least 50%? |
| 4 | Were all the subjects selected or recruited from the same or similar populations (including the same time period)? Were inclusion and exclusion criteria for being in the study prespecified and applied uniformly to all participants? |
| 5 | Was a sample size justification, power description, or variance and effect estimates provided? |
| 6 | For the analyses in this paper, were the exposure(s) of interest measured prior to the outcome(s) being measured? |
| 7 | Was the timeframe sufficient so that one could reasonably expect to see an association between exposure and outcome if it existed? |
| 8 | For exposures that can vary in amount or level, did the study examine different levels of the exposure as related to the outcome (e.g., categories of exposure, or exposure measured as continuous variable)? |
| 9 | Were the exposure measures (independent variables) clearly defined, valid, reliable, and implemented consistently across all study participants? |
| 10 | Was the exposure(s) assessed more than once over time? |
| 11 | Were the outcome measures (dependent variables) clearly defined, valid, reliable, and implemented consistently across all study participants? |
| 12 | Were the outcome assessors blinded to the exposure status of participants? |
| 13 | Was loss to follow-up after baseline 20% or less? |
| 14 | Were key potential confounding variables measured and adjusted statistically for their impact on the relationship between exposure(s) and outcome(s)? |
| **Before-After (Pre-Post) Studies With No Control Group** | |
| **Number** | **Criteria** |
| 1 | Was the study question or objective clearly stated? |
| 2 | Were eligibility/selection criteria for the study population prespecified and clearly described? |
| 3 | Were the participants in the study representative of those who would be eligible for the test/service/intervention in the general or clinical population of interest? |
| 4 | Were all eligible participants that met the prespecified entry criteria enrolled? |
| 5 | Was the sample size sufficiently large to provide confidence in the findings? |
| 6 | Was the test/service/intervention clearly described and delivered consistently across the study population? |
| 7 | Were the outcome measures prespecified, clearly defined, valid, reliable, and assessed consistently across all study participants? |
| 8 | Were the people assessing the outcomes blinded to the participants' exposures/interventions? |
| 9 | Was the loss to follow-up after baseline 20% or less? Were those lost to follow-up accounted for in the analysis? |
| 10 | Did the statistical methods examine changes in outcome measures from before to after the intervention? Were statistical tests done that provided p values for the pre-to-post changes? |
| 11 | 11. Were outcome measures of interest taken multiple times before the intervention and multiple times after the intervention (i.e., did they use an interrupted time-series design)? |
| 12 | 12. If the intervention was conducted at a group level (e.g., a whole hospital, a community, etc.) did the statistical analysis take into account the use of individual-level data to determine effects at the group level? |
| Taken from: <https://www.nhlbi.nih.gov/health-topics/study-quality-assessment-tools> | |

| **S4 Table. Relevant conference abstract excluded from synthesis** |
| --- |
| Relevant Conference Abstract Excluded |
| Arighna Mukherjee, Arghya Majumdar. A clinical profile of patients with obstructive sleep apnea and end stage renal disease on haemodialysis. Indian Journal of Nephrology. Abstract No 116, 15^th^ December 2023 |
